# Infiltration-RNAseq Reveals Enhanced Defense Responses in *Nicothiana benthamiana* Leaves Overexpressing the Banana Gene *MaWRKY45*

**DOI:** 10.3390/plants14030483

**Published:** 2025-02-06

**Authors:** Sergio García-Laynes, Carlos Ligne Calderón-Vázquez, Carlos Puch-Hau, Virginia Aurora Herrera-Valencia, Santy Peraza-Echeverria

**Affiliations:** 1 Unidad de Biotecnología, Centro de Investigación Científica de Yucatán, Calle 43 No. 130 x 32 y 34, Colonia Chuburná de Hidalgo, Mérida 97205, Yucatán, Mexico; sergio.laynes@cicy.mx; 2 Instituto Politécnico Nacional, Centro Interdisciplinario de Investigación para el Desarrollo Integral Regional CIIDIR Unidad Sinaloa, Guasave 81100, Sinaloa, Mexico; ccalderon@ipn.mx; 3 Tecnológico Nacional de México, Campus Instituto Tecnológico Superior de Valladolid, Carretera Valladolid-Tizimín, km 3.5, C.P., Valladolid 97780, Yucatán, Mexico; cashi026@hotmail.com

**Keywords:** *Nicotiana benthamiana*, banana, *MaWRKY45*, Infiltration-RNAseq method, plant immunity

## Abstract

The banana gene *MaWRKY45* gene encodes a WRKY transcription factor (TF) that is closely related to *OsWRKY45*, which is a master regulator of defense responses in rice. MaWRKY45 is a transcription factor with proven transactivation activity and nuclear localization. Its expression is upregulated by the defense phytohormones salicylic acid (SA) and jasmonic acid (JA). Despite these findings, its transcriptome-wide impact during overexpression remains unexplored. Accordingly, the present study employed the Infiltration-RNAseq method to identify differentially expressed genes (DEGs) resulting from the overexpression of *MaWRKY45* in the leaves of the model plant *Nicotiana benthamiana*. A total of 2473 DEGs were identified in *N. benthamiana* leaves overexpressing the banana gene *MaWRKY45*. Of these, 1092 were up-regulated and 1381 were down-regulated. Among the genes that were found to be up-regulated, those encoding proteins that are involved in plant immunity were identified. These included disease resistance receptors, proteins that are involved in cell wall reinforcement, proteins that possess antimicrobial and insecticidal activities, and defense-related TFs. It was thus concluded that the function of the banana gene *MaWRKY45* is associated with the plant immune system, and that its overexpression can lead to enhance defense responses.

## 1. Introduction

The banana is a staple food for millions of people in the tropics and is one of the most prolifically produced fruits in the world, with an estimated 170 million tons produced annually. Its estimated value is over USD 51 billion [1], making it a significant source of foreign currency for many countries. Furthermore, this fruit crop represents a significant source of foreign currency for the leading exporters, with approximately 15% of production traded internationally, valued at an estimated USD 13.6 billion [1]. The majority of bananas produced are consumed locally, thereby ensuring food security for millions of people in developing countries. Fungal diseases represent a significant challenge to global banana production. Among these, black Sigatoka, caused by the airborne pathogen *Pseudocercospora fijiensis*, is particularly destructive to susceptible cultivars, leading to leaf necrosis and premature fruit ripening. At present, the primary method for controlling this pathogen in commercial plantations is through the extensive use of fungicide applications, which has the dual effect of increasing production costs and contributing to environmental pollution. A more serious fungal threat is Fusarium wilt, which is caused by the soil-borne pathogen *Fusarium oxysporum* f. sp. *cubense* (Foc). This pathogen infects the roots of susceptible cultivars, causing lethal vascular wilt [2,3]. The strategy of controlling this pathogen in the field with fungicides has proven to be ineffective. The emergence of Foc Tropical Race 4 (TR4) has resulted in a pandemic situation, placing the food security of millions of people at significant risk. It is therefore imperative to identify a more effective and sustainable method of controlling these pathogens in the field, such as genetic resistance. In this regard, the genomes of numerous wild bananas that exhibit resistance to the most detrimental diseases affecting banana cultivars have been sequenced [4,5,6], thereby facilitating the study of gene families involved in plant immunity and accelerating the selection of candidate genes for genetic improvement programs. Research into enhancing banana resistance to fungal pathogens through genetic transformation has been reported [7,8]. However, it is essential to identify additional genes involved in plant immunity in order to expand the repertoire of biotechnological tools necessary to combat the aggressiveness and rapid evolution of these fungal threats.

WRKY transcription factors (TFs) are key regulators of plant immune responses, binding to cis-regulatory elements in gene promoters to activate or repress transcription. These proteins are part of a large plant-specific gene family, characterized by a conserved WRKY domain (~60 amino acids) at the N-terminus and a zinc-finger motif at the C-terminus [9]. WRKY transcription factors bind to the W-box cis-acting element, which is composed of the sequence TTGACY, in the promoters of their target genes. The W-boxes are pervasive in the promoter regions of stress-inducible genes [10,11]. Based on the number of WRKY domains and the type of zinc finger motif present, three major groups of WRKY proteins have been identified [12]. Group I comprises two WRKY domains in tandem and a C2H2 zinc finger, whereas groups II and III contain only one WRKY domain with either a C2H2 zinc finger (group II) or a C2HC zinc finger (group III). Overexpression of WRKY genes has been demonstrated to confer disease resistance against a broad range of pathogens [9,13], positioning them as a promising class of TFs for engineering broad-spectrum resistance in crops. One of the most promising WRKY candidates in monocotyledonous (monocot) crops is the rice *OsWRKY45*. Overexpression of this gene in transgenic rice resulted in increased resistance against both bacterial and fungal pathogens [14,15]. However, constitutive overexpression of *OsWRKY45* caused adverse effects on growth and yield [14,15]. The overexpression of this TF was optimized in transgenic rice by utilizing either a moderate constitutive promoter or a pathogen-inducible promoter, resulting in rice plants without adverse effects on growth and development [16,17]. These findings have initiated a novel area of research in WRKY bioengineering, with the objective of enhancing the immune system in crops of significant importance to food security, while avoiding any adverse effects on agronomic traits.

A recent study has identified a potential orthologue of *OsWRKY45* in a wild banana (*Musa acuminata* ssp. *malaccensis*), which has demonstrated resistance to the most damaging pathogens of commercial bananas. This gene has been designated *MaWRKY45*, reflecting its close phylogenetic relationship with *OsWRKY45* [18]. Both MaWRKY45 and OsWRKY45 are classified as group III WRKY TFs. Additionally, the MaWRKY45 protein was observed to localize to the nucleus of onion epidermal cells and demonstrate transcription activity in yeast cells. Notably, the *MaWRKY45* gene exhibited increased expression in response to the phytohormones salicylic acid (SA) and jasmonic acid (JA) in banana plants. Both SA and JA play a pivotal role in immune responses, including systemic acquired resistance (SAR) and induced systemic resistance (ISR), respectively [19].

To gain new insights into the function of a TF, the strategy of overexpression in transgenic plants has proven useful in gathering information about the genes that act downstream of a particular TF [20]. Nevertheless, this approach is laborious and time-consuming in the context of tropical fruit crops. It can take up to 15 months to obtain stable transgenic plants in banana [21]. Furthermore, the overexpression of TFs in transgenic plants can result in adverse effects on growth and development, which can delay the in vitro regeneration process [22]. A novel approach, termed Infiltration-RNAseq, has recently been developed, which combines the rapid and straightforward agroinfiltration technique with high-throughput RNA sequencing technology. This method has been employed for the profiling of transcription factor-mediated gene expression changes [23]. The proof of concept for the Infiltration-RNAseq method was demonstrated using a *Medicago truncatula* MYB TF gene (*MtLAP1*) involved in the regulation of the anthocyanin pathway. This gene was transiently overexpressed in the leaves of *M. truncatula* plants, and their transcriptome was subsequently analyzed. Leaves agroinfiltrated with *MtLAP1* exhibited red coloration, indicative of the production of the anthocyanin pigment. The transcriptome profile demonstrated the upregulation of numerous genes encoding enzymes and transcription factors involved in the anthocyanin biosynthetic pathway. Additionally, the Infiltration-RNAseq method was employed in the model plant *Nicotiana benthamiana* to transiently overexpress TF genes from other plant species, including *AcMYB10* and *AtLEC2* from kiwi fruit and *Arabidopsis*, respectively. The Infiltration-RNAseq method was completed in a few days in both *M. truncatula* and *N. benthamiana*, a significant reduction in time compared to the months typically required for stable genetic transformation. This approach has the potential to accelerate the functional analysis of other classes of transcription factors [23]. The utility of *N. benthamiana* in providing molecular and cell biological support to less tractable systems is unprecedented in plant biology [24]. *N. benthamiana* has been used to characterize proteins from many agronomically important monocots (including banana) or dicots (dicotyledonous plants). The use of *N. benthamiana* covers a wide range of applications, including protein localization, protein expression (such as vaccine antigens or antibodies), protein–protein interactions, protein purification, gene silencing and, most recently, infiltration RNA-seq [23,24].

In this study, we employed the Infiltration-RNAseq method to transiently overexpress the banana gene *MaWRKY45* in *N. benthamiana* leaves, thereby enabling the identification of transcription factor-mediated transcriptional changes. The analysis yielded insights into the involvement of multiple genes in plant biotic stress responses related to immune signaling, cell wall reinforcement, and antimicrobial activity. These findings indicate that *MaWRKY45* plays a pivotal role in regulating the banana immune response and offers novel insights into the transcriptional landscape orchestrated by a defense-related WRKY gene. Therefore, *MaWRKY45* is a promising TF for bioengineering disease resistance in banana cultivars against the primary fungal pathogens *P. fijiensis* and Foc, as well as in other staple crops facing similar threats. This could ultimately lead to a significant reduction in crop losses caused by plant pathogens.

## 2. Results

### 2.1. Analysis of N. benthamiana De Novo Reference Transcriptome Under Banana MaWRKY45 Overexpression

We obtained high-quality total RNA, confirmed by robust quality control metrics (see Table S1), ensuring integrity and suitability for downstream applications. The sequencing libraries passed stringent quality assessments (see Table S2), resulting in a total of 307,309,400 high-quality raw reads (see Table S3) from six libraries (*MaWRKY45* overexpression and mock, each with three biological replicates). The assembly statistics revealed a N50 contig of 1289 and an average contig size of 782 (Table 1). A total of 189,020 contigs were assembled, of which 167,935 were identified as unigenes and selected for further analysis. Additionally, 24,853 unigenes with open reading frames (ORFs) were identified.

### 2.2. Global DEG Analysis of N. benthamiana Leaves Overexpressing MaWRKY45

**Figure 1:**
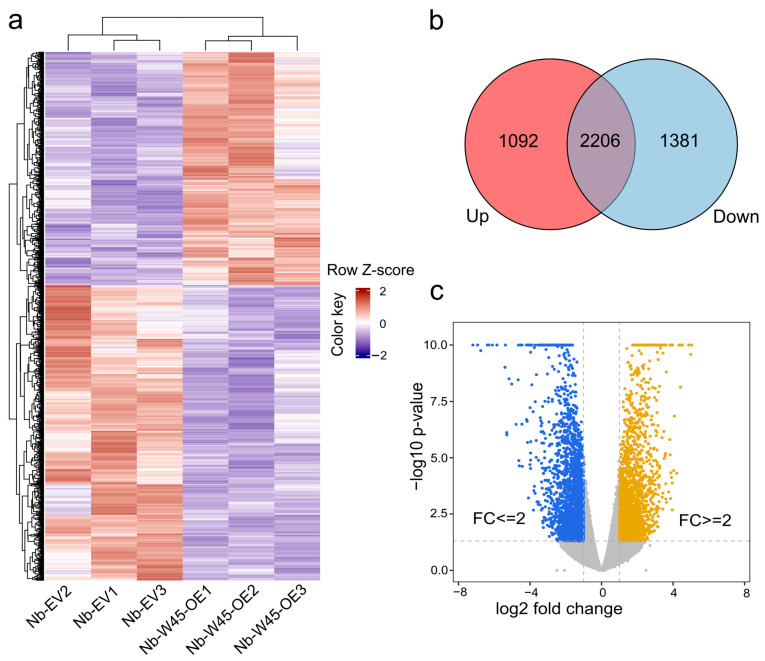
Differentially expressed genes (DEGs) in *N*. *benthamiana* leaves overexpressing banana *MaWRKY45*. (**a**) Heatmap of 2473 DEGs identified with a fold change (FC) ≥ 2 and a Benjamini–Hochberg FDR-adjusted *p*-value < 0.05. Up- and down-regulated genes are shown in red and blue, respectively. (**b**) Venn diagram highlighting the distribution of DEGs with FC ≥ 2 and raw *p*-values. (**c**) Volcano plot depicting the −log10 *p*-value against log2 FC, illustrating the significance and magnitude of differential expression. Yellow points represent DEGs with FC ≥ 2 and a Benjamini–Hochberg FDR-adjusted *p*-value < 0.05, while blue points correspond to DEGs with FC ≤ 2 and a Benjamini–Hochberg FDR-adjusted *p*-value < 0.05. Grey points represent genes with FC ≤ 2 and *p*-values < 0.05. The dashed line indicates the threshold for statistical significance. Nb-W45-OE1-3, treatment samples; Nb-EV1-3, mock samples.

To identify DEGs, we conducted a comparative analysis of the expression profiles between *MaWRKY45* overexpression samples and mock samples. A total of 2473 genes were identified as significantly expressed with a fold change of ≥2 and a Benjamini–Hochberg FDR-adjusted *p*-value of <0.05. Of these, 1092 DEGs demonstrated up-regulation, while 1381 exhibited down-regulation in their expression (Figure 1a,b).

**Figure 2:**
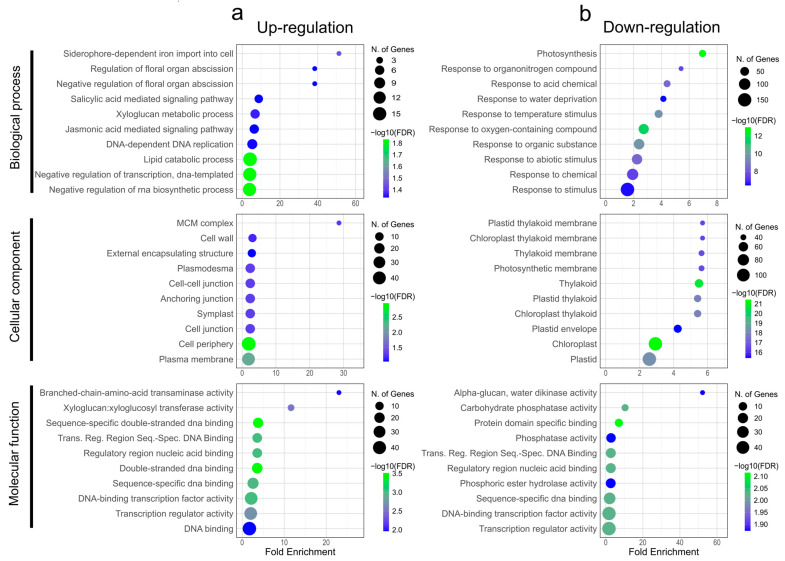
Gene ontology (GO) enrichment analysis of differentially expressed genes (DEGs) in *N*. *benthamiana* leaves overexpressing *MaWRKY45*. (**a**) Top 10 GO terms for up-regulated DEGs, categorized into biological processes, cellular components, and molecular functions. (**b**) Corresponding GO terms for down-regulated DEGs. Dot size represents the number of genes annotated to each term, and color intensity reflects statistical significance.

Gene ontology (GO) enrichment analysis of the DEGs (Figure 2) identified the top ten GO terms for biological processes, cellular components, and molecular functions, providing insights into the roles of these genes in stress responses. Notably, in the biological process category, GO terms associated with biotic stress response, such as SA and JA signaling pathways, were identified in the up-regulated DEGs (Figure 2a). In contrast, in the down-regulated DEGs, GO terms related to abiotic and development were favored (Figure 2b).

**Figure 3:**
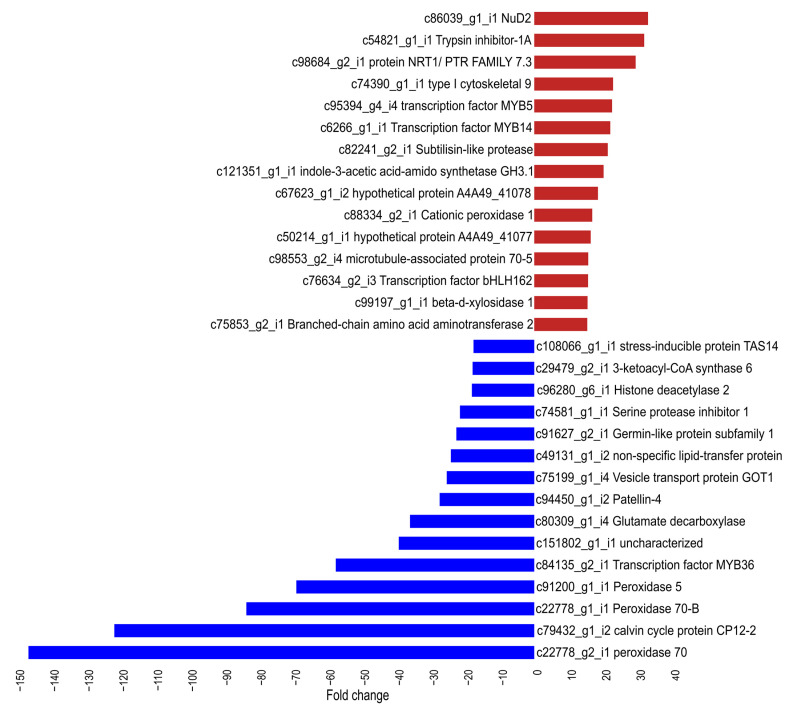
The 15 most significantly up- or down-regulated genes, as determined by fold change, are. Red bars indicate positive fold changes (up-regulation), while blue bars indicate negative fold changes (down-regulation). The gene IDs of *N. benthamiana* and the gene names of its homologs are provided for reference.

In the cellular component category, membrane-related GO terms were found to be significantly enriched among the up-regulated genes (Figure 2a), whereas photosynthetic-related GO terms were enriched among the down-regulated genes (Figure 2b). With regard to molecular function, DNA binding was the most significantly enriched GO term for both the up-regulated and down-regulated sets of DEGs (Figure 2a,b). In the cellular component category, membrane-related GO terms were found to be significantly enriched among the up-regulated genes (Figure 2a), whereas photosynthetic-related GO terms were enriched among the down-regulated genes (Figure 2b). With regard to molecular function, DNA binding was the most significantly enriched GO term for both the up-regulated and down-regulated sets of DEGs (Figure 2a,b). It is noteworthy that among the most highly expressed genes, there was a notable prevalence of genes encoding proteins involved in biotic stress responses. For example, homologs of the Nudix hydrolase 2 (NbNud2), trypsin inhibitor-1A, NRT1/PTR family 7.3, MYB5, and MYB14 were identified. Conversely, the most significantly down-regulated genes included those encoding proteins involved in oxidative stress responses, such as peroxidase 70, peroxidase 70-B, peroxidase 5, MYB36, and Patellin 4 (Figure 3).

### 2.3. Transcriptional Regulation of Biotic Stress Responses in N. benthamiana Leaves Overexpressing MaWRKY45

**Figure 4:**
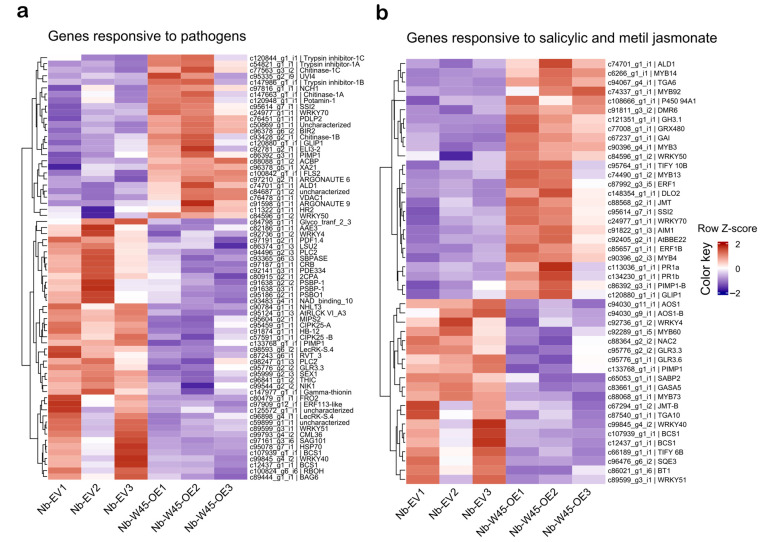
Differentially expressed genes (DEGs) related to biotic stress response. (**a**) Hierarchical clustering of annotated DEGs responsive to phytopathogens (bacteria, fungi, and viruses). (**b**) DEGs responsive to the phytohormones SA and JA. The data were hierarchically clustered using the Euclidean method (complete linkage), organizing the data based on contig and sample similarity with normalized expression levels. The color scale shows the gene expression range while the red and blue color bars represent up- and down-regulated genes, respectively. Nb-W45-OE1-3, treatment samples; Nb-EV1-3, mock samples.

A data-mining analysis of previously annotated genes was conducted to identify DEGs involved in biotic stress responses. Firstly, a search was conducted for genes responsive to bacteria, fungi, and viruses (Figure 4a). A total of 40 DEGs responsive to bacteria were identified, comprising 13 up-regulated and 27 down-regulated genes. A total of 31 DEGs responsive to fungi were identified, comprising 13 up-regulated and 18 down-regulated genes. Regarding viruses, only 10 genes were identified as responsive, comprising four up-regulated and six down-regulated genes. The WRKY transcription factor family is the most overrepresented among genes responsive to bacteria, fungi, and viruses. For instance, the expression profiles of *WRKY40*, *WRKY50* and *AtWRKY70*, which are plant immune-responsive genes [9], exhibit similarities between the bacterial and fungal sets. Furthermore, some membrane receptors were identified, including FLS2, Xa21, and PIMP1, which belong to the LRR-RLK family and can recognize microbial-associated molecular patterns (MAMPs). Furthermore, the homolog of *RPW8.2* (*HR2*), which belongs to the NBS-LRR family, was found to be highly expressed, with a 14-fold increase. In the set of genes responsive to fungi, four potato inhibitors belonging to the proteinase inhibitor family were identified as being up-regulated. It is noteworthy that the gene trypsin inhibitor-1A exhibited a markedly elevated expression level in *N. benthamiana* leaves overexpressing *MaWRKY45*, with a fold change of 31. Additionally, genes encoding antimicrobial peptides (chitinases and pathogenesis related proteins) and enzymes involved in the biosynthesis of protective polymers, such as the cuticle (ELI-3-2) and lignin (ACBP), were identified as being up-regulated. Among the genes identified in response to viral infection were *Argonaute 6*, *Argonaute 9*, *PDLP2*, and *SSI2*.

Moreover, a search for DEGs responsive to the defense phytohormones SA and JA was conducted (Figure 4b). Twenty-three DEGs responsive to SA were identified, of which 12 were up-regulated and 11 were down-regulated. Similarly, 31 genes were found to be responsive to JA, comprising 18 up-regulated and 13 down-regulated genes. Among the transcription factors identified, those belonging to the WRKY, ERF, and MYB families were found to be responsive to both SA and JA, with the MYB members exhibiting the highest level of up-regulation. It was observed that this category of genes, which are responsive to phytohormones, exhibited similarities to a subset of genes that are responsive to microorganisms (Figure 4a). This subset included the up-regulated genes *WRKY50*, *WRKY70*, *ALD1*, *SSI2*, and *GLIP1*, which have been previously described as pathogen-induced genes. Additionally, we identified genes associated with SAR, including the molecular markers Pathogenesis-Related protein 1 (PR1) and Downy Mildew Resistance 6 (DMR6). It is noteworthy that some of the down-regulated genes in this category are also associated with abiotic stress (*MYB60*, *MYB73*, and *GASA5*) and plant development (*BT1*). Interestingly, we observed that several genes involved in ISR defense or JA biosynthesis (*AOS1*, *GLR3.6*, *TIFY 6B*, *JMT*, and *SQE3*) exhibited down-regulation.

### 2.4. MaWRKY45 Overexpression in N. benthamiana Leaves Induces Transcriptional Changes in Multiple Transcription Factor Genes

**Figure 5:**
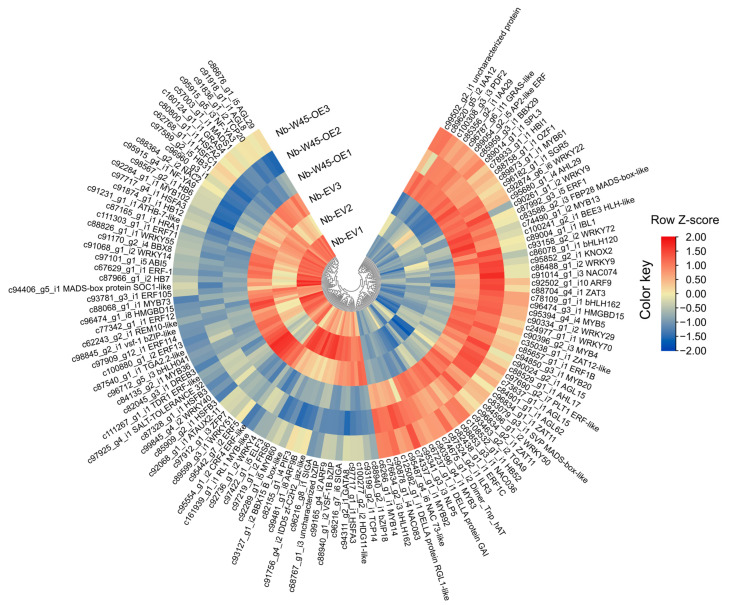
Hierarchical clustering of 127 differentially expressed genes encoding transcription factors from *N*. *benthamiana*, comparing treatment samples (Nb-W45-OE1-3) with mock samples (Nb-EV1-3). The color scale shows the gene expression range; the red and blue color bars represent up- and down-regulated genes, respectively.

**Figure 6:**
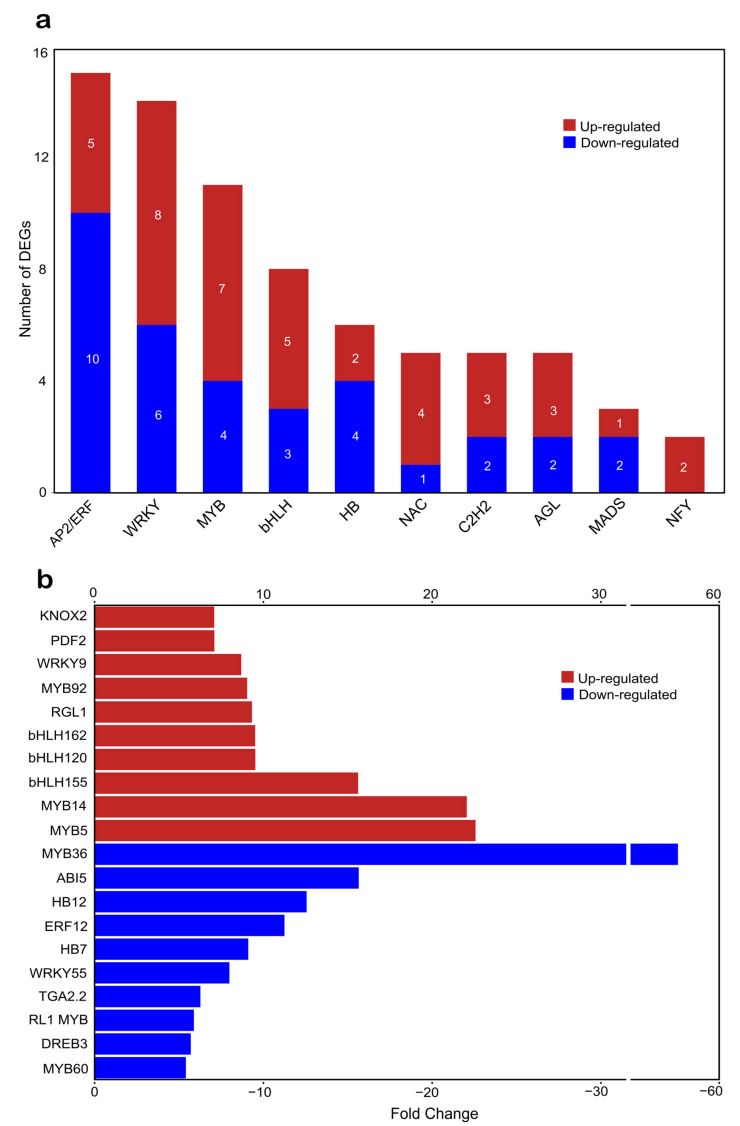
The most overrepresented families and members of transcription factors (TFs) differentially expressed in *N*. *benthamiana* leaves overexpressing the banana *MaWRKY45* gene. (**a**) The top 10 families with the highest number of members are shown. Bars in red and blue represent up- and down-regulated genes, respectively. (**b**) The top ten TF genes with the highest fold changes in either up- or down-regulation are plotted. Bars in red and blue indicate up- and down-regulated genes, respectively.

**Figure 7:**
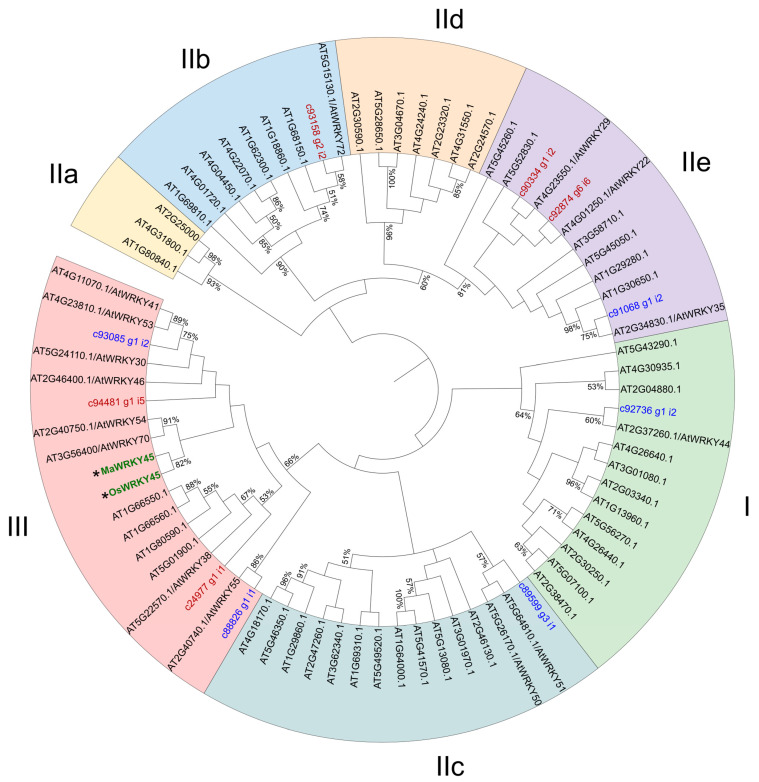
Phylogenetic tree of the amino acid sequences of 10 WRKY-DEGs from *N. benthamiana* leaves overexpressing the banana *MaWRKY45* gene, as well as the complete *Arabidopsis* WRKY family, plus MaWRKY45 (GenBank accession number OP186310) and OsWRKY45 (GenBank accession number AK066255). The sequences were aligned using the MUSCLE algorithm, and the tree was constructed using the neighbor-joining method. Numbers on the branches indicate the percentage of 1000 bootstrap replications supporting the particular nodes and only those ≥50% are shown. The groups and subgroups of WRKY TFs are highlighted in different colors. The *N. benthamiana* up-regulated WRKY genes are highlighted in red, while down-regulated WRKY genes are highlighted in blue. The MaWRKY45 and OsWRKY45 sequences are colored in green and highlighted with asterisks.

**Figure 8:**
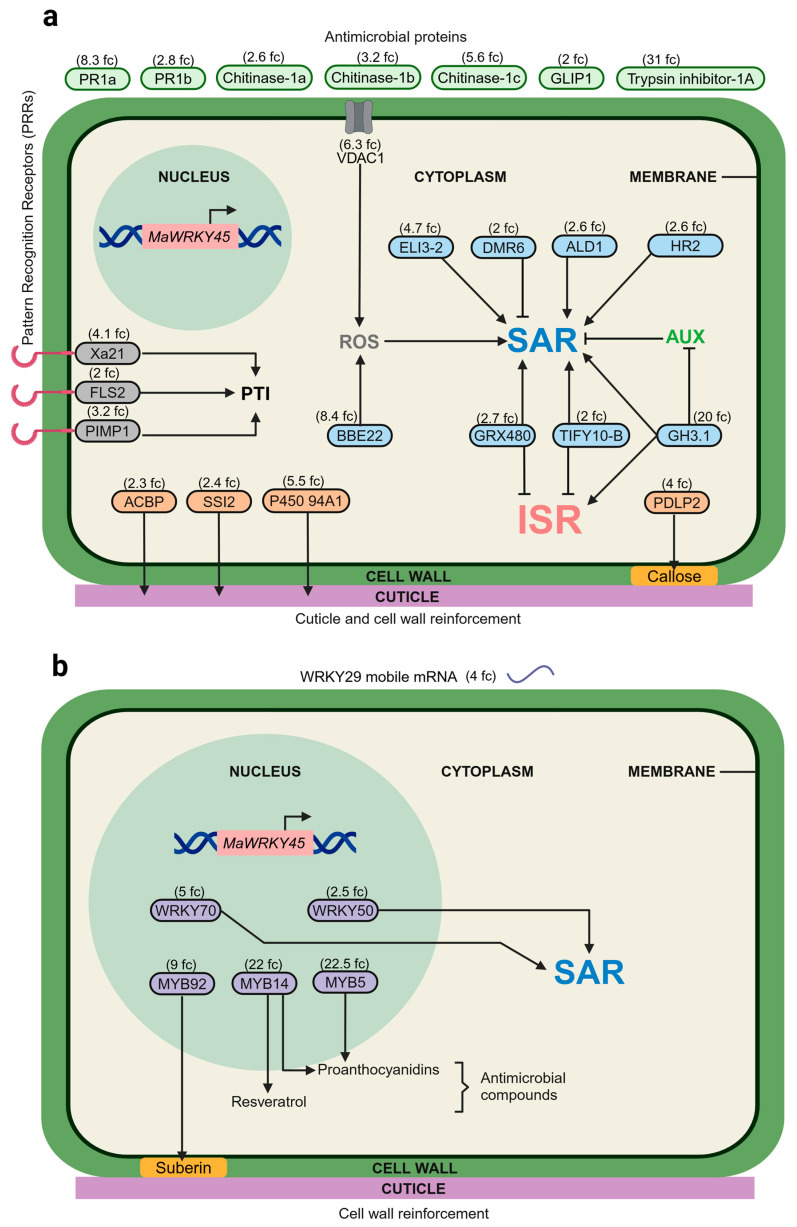
Overexpression of banana *MaWRKY45* activates a biotic defense regulatory network in *N*. *benthamiana*. (**a**) Extracellular, membrane and cytoplasmic proteins related to biotic stress responses encoded by genes up-regulated by *MaWRKY45* overexpression. (**b**) Transcription factors involved in biotic stress responses encoded by genes up-regulated by *MaWRKY45* overexpression. Proteins with cytoplasmic functions are shown in blue, proteins associated with membrane functions are shown in gray, proteins associated with cell wall and cuticle biosynthesis are shown in orange, proteins with extracellular functions are shown in green, and transcription factors are shown in purple. Arrows indicate positive regulation, while inhibition lines indicate negative regulation. The fold change values are shown in parentheses. ACBP, Acyl-CoA-Binding Protein; ALD1, AGD2-Like Defense Response Protein 1; AUX, auxins; BBE22, Berberine Bridge Enzyme-like 22; DMR6, Downy Mildew Resistant 6; ELI3-2, Early Lipoxygenase Inducible protein 3-2; FLS2, Flagellin-Sensing 2; GH3.1, Gretchen Hagen 3.1; GLIP1, GDSL Lipase 1; GRX480, Glutaredoxin 480; HR2, homolog of RPW8.2; ISR, Induced Systemic Resistance; MaWRKY45, *Musa acuminata* WRKY transcription factor gene; MYB5, myeloblastosis transcription factor 5; MYB14, myeloblastosis transcription factor 14; MYB92, myeloblastosis transcription factor 92; P450 94A1, Cytochrome P450 94A1; PDLP2, Plasmodesmata-Located Protein 2; PIMP1, Pathogen-Induced Membrane Protein 1; PR1a, Pathogenesis-Related Protein 1a; PR1b, Pathogenesis-Related Protein 1b; PTI, Pattern-Triggered Immunity; ROS, Reactive Oxygen Species; SAR, Systemic Acquired Resistance; SSI2, Suppressor of Salicylic Acid Insensitivity 2; TIFY10-B, member of the TIFY family; VDAC1, Voltage-Dependent Anion Channel 1; WRKY29, WRKY transcription factor 29; WRKY50, WRKY transcription factor 50; WRKY70, WRKY transcription factor 70; Xa21, Xanthomonas resistance protein 21.

The identification of differentially expressed TFs was conducted by searching for DNA-binding domains in the PFAM database, which was generated from the data on DEGs. A total of 127 differentially expressed TFs were identified, representing 5% of the total DEGs. Of these, 67 were up-regulated and 60 were down-regulated (Figure 5). The majority of these transcription factor genes (74) were classified into 10 major families: *AP2/ERF* (15 DEGs), *WRKY* (14), *MYB* (11), *bHLH* (8), *HB* (6), *NAC* (5), *C2H2* (5), *AGL* (5), *MADS* (3), and *NFY* (2) (Figure 6a). The remaining 53 TFs are distributed across a wide variety of TF families. The most overrepresented families were *AP2/ERF*, *WRKY*, and *MYB*, which collectively represented 33% of the 127 differentially expressed TFs. The five most highly expressed TFs were *MYB5*, *MYB14*, *bHLH155*, *bHLH120*, and *bHLH162*. Conversely, the five most repressed TFs were *MYB36*, *ABI5*, *HB12* (Homeobox 12), *ERF12*, and *HB7* (Homeobox 7) (Figure 6b). A phylogenetic analysis of 10 differentially expressed WRKY genes from *N. benthamiana* revealed that the genes were distributed across all three groups and subgroups of the WRKY TF classification pattern (Figure 7). The most overrepresented group was Group III, which included three members that were differentially expressed. These results provide a comprehensive overview of how the *MaWRKY45* gene coordinates transcriptional dynamics, likely through direct and indirect regulation of other TFs, thereby amplifying its regulatory scope. In this regard, we constructed a transcriptional network model based on DEGs related to biotic stress responses in plants, including TFs (Figure 8). This model comprises genes found to be up-regulated and implicated in plant immunity responses.

### 2.5. RT-qPCR Analysis of Genes Associated with MaWRKY45 Overexpression

**Figure 9:**
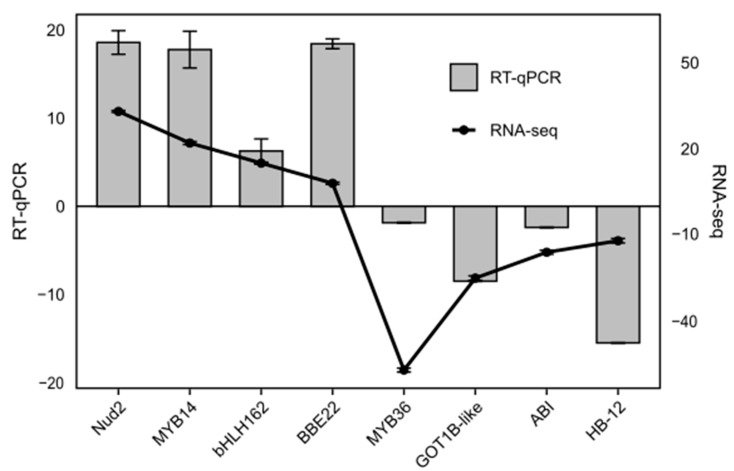
Validation of the RNA-seq data conducted through an RT-qPCR assay. The comparative expression profile of RNA-seq and RT-qPCR results of eight differentially expressed genes (DEGs) from *N. benthamiana* leaves (four up-regulated and four down-regulated) overexpressing the coding sequence of the banana *MaWRKY45* gene is presented. The experimental assay was repeated, yielding comparable results. RT-qPCR relative expression values were calculated using the 2^−ΔΔCt^ method [25]. The mean ± S.D. of three biological replicates is presented. The line represents the expression level of the eight DEGs transformed into fold change values. The bars represent the expression level of the RT-qPCR results.

Eight DEGs associated with the transient overexpression of the banana gene *MaWRKY45* in *N. benthamiana* leaves were selected for validation of the RNA-seq assay (four up-regulated and four down-regulated genes). These genes were chosen from a list of the most differentially expressed genes identified (Table S4). The RT-qPCR and RNA-seq assays showed a comparable expression profile for all eight selected genes (Figure 9). This outcome corroborates the expression data obtained through the Infiltration-RNA-seq method.

## 3. Discussion

Gene expression is a fundamental process that drives plant growth, development, and interaction with the surrounding environment. TFs play a pivotal role in the gene transcription process. They regulate the spatial and temporal expression of genes in a coordinated manner. Similarly, plant response to biotic stress is also subject to regulation by TFs. Some TFs are considered master regulators, acting at the top of a regulatory hierarchy and determining a complete defense response program [26]. Notable examples include the NPR1 protein in the SAR pathway in dicot plants [27] and OsWRKY45 in monocot plants [28]. The banana gene *MaWRKY45* encodes a WRKY transcription factor belonging to group III, which is induced by SA and MeJA [18]. This gene is a potential ortholog of rice *OsWRKY45*, which has been demonstrated to regulate defense responses independently of OsNHI (an NPR1 ortholog). In this study, we employed the Infiltration-RNAseq method [23] to investigate *MaWRKY45*-mediated transcriptional changes in the model plant *N. benthamiana*. In contrast to the findings of Bond et al. [23], who reported a range of 7–13 million clean reads per library, our further sequencing efforts yielded approximately 45–55 million clean reads per library, resulting in over six times the data. This may partly explain the significantly higher number of total DEGs observed in our study, with 2473 compared to the 118 reported by Bond et al. [23]. Furthermore, Bond et al. [23] focused on MaLAP1, a TF that is specifically involved in regulating the anthocyanin pathway, which involves a relatively limited number of genes [29]. In contrast, stress defense responses are known to induce substantial changes in transcriptional dynamics, which result in a temporary cessation of plant development and growth in order to mitigate the damage caused by stress [30,31]. It can be reasonably deduced that TFs acting as master regulators in plant defense may cause significant transcriptomic changes.

The quality of the assembled contigs was validated by several metrics, including N50 (1289 bp) and average contig length (782 bp) (Table 1). A de novo transcriptome assembly of *N. benthamiana* leaves under *MaWRKY45* overexpression was generated using these data. A total of 2473 DEGs were identified with a fold change of ≥2 and a false discovery rate (FDR)-adjusted *p*-value of <0.05, which is consistent with the expected range of DEGs reported in similar studies. For example, the overexpression of the maize gene *ZmBZR1* TF, which is known to regulate organ development and seed size, resulted in the differential expression of 1380 genes with a sequencing depth of 20 million reads per library [32]. In our study, the majority of DEGs exhibited downregulation (1381) compared to those that were upregulated (1092), indicating the suppression of several biological processes while others were activated (Figure 1). Recent evidence has highlighted the dual role of TFs, which can either repress or activate the transcription of downstream target genes. For example, the *Arabidopsis* TGA2 transcription factor is necessary for the repression of *PR-1* [33], but it can also activate the transcription of *PR-1* in an SA- and NPR1-dependent manner [34]. A recent study employing chromatin immunoprecipitation sequencing (ChIP-seq) demonstrated that the *Arabidopsis* WRKY18, WRKY40, and WRKY33 TFs each bind to over 1000 loci, predominantly at W-box elements within the 500 bp promoter regions of their target genes [35]. These findings provide an explanation for the significant changes in transcriptional profiles observed in our study resulting from the overexpression of the banana *MaWRKY45* gene.

The GO enrichment analysis revealed a significant enrichment in SA and JA signaling biological processes, membrane-associated components, and DNA-binding activity functions among the up-regulated genes (Figure 2). The interplay between the synergistic and antagonistic effects of the JA and SA signaling pathways is known to equip plants with the regulatory capacity to withstand biotic stress [36,37]. Furthermore, the significance of the cell wall, which serves as a physical barrier to restrict pathogen entry, was also highlighted [38]. The up-regulation of DNA-binding activity indicates the activation of defense mechanisms essential for combating pathogen-induced diseases [39]. These findings, in conjunction with the observation that a considerable number of genes involved in photosynthesis were down-regulated in response to *MaWRKY45* overexpression, are in alignment with the results of the meta-analysis conducted by Zhang et al. [40]. This analysis reported that SA and JA signaling pathways co-induce a broad spectrum of disease responses, co-repress genes related to photosynthesis, and reallocate resources from growth toward defense.

To elucidate the DEGs involved in the response to biotic stress, we conducted a search for genes responsive to pathogens, including bacteria, fungi, and viruses, as well as responsive to SA and JA phytohormones (Figure 4). The analysis yielded a multitude of DEGs associated with diverse processes, including cell wall reinforcement, receptor activity, signal transduction, signal amplification, transcription factor regulation, and defense responses. Specifically, we identified genes that were up-regulated and involved in cell wall reinforcement, including *PDLP2*, *P450 94A1*, *ACBP*, and *SSI2*. These genes contribute to plant resistance against pathogens by aiding in the biosynthesis of the cuticle and lignin, as well as callose deposition [41,42,43]. Moreover, we observed the up-regulation of genes encoding receptors, including LRR-RLK *FLS2*, *Xa21*, and *PIMP1*, which are implicated in defense mechanisms through the recognition of MAMPs [39,44,45]. Furthermore, the gene for co-receptor BIR2 (receptor-like kinase), which is associated with fungal defense responses [46], was also found to be up-regulated. Furthermore, the NBS-LRR receptor gene *HR2* (homolog of *RPW8.2*) was found to be up-regulated, as it is involved in defense responses against fungal pathogens and provides resistance to powdery mildew [47]. These findings suggest that *MaWRKY45* may regulate the expression of immune receptor genes involved in both pattern-triggered immunity (PTI) and effector-triggered immunity (ETI) defense response systems. Additionally, we observed a notable reduction in the expression of peroxidase-like genes for peroxidase 70, the peroxidase 70-B isoform, and peroxidase 5 (Figure 3). These genes are known to play a role in the detoxification of cellular reactive oxygen species (ROSs), which are compounds involved in signaling and linked to biotic stress responses [48]. The suppression of these genes suggests that the overexpression of *MaWRKY45* may promote ROS production and signal transduction, thereby amplifying the cellular response to biotic stress. Overexpression of WRKY genes in transgenic *Arabidopsis*, poplar and pepper has also resulted in defense gene expression changes and enhance disease resistance [9,13].

Furthermore, we identified the up-regulation of a glutathione S-transferase (GST) gene (*P450 94A1*), the enzyme of which plays a role in cellular detoxification [14]. Similarly, *OsWRKY45* has been demonstrated to induce the expression of glutathione S-transferase in rice [14]. In this regard, *MaWRKY45* and *OsWRKY45* exhibit analogous regulatory patterns with respect to the redox balance. Additionally, genes encoding peptides with antimicrobial activity, such as *GLIP1*, were also found to be up-regulated. In *Arabidopsis*, *AtGLIP1* plays a role in defense pathways that directly target the cell wall of *Alternaria brassicicola* [49]. In *Capsicum annuum*, it contributes to resistance against mechanical stress by modulating the expression of *CaPR-4* [50]. Furthermore, a trypsin inhibitor-1A gene exhibited a notable increase in transcription, with a fold change of 31 (see Figure 3 and Figure 4). The protein encoded by this class of genes has been demonstrated to exhibit both insect-deterrent and antimicrobial activities in tobacco and soybean [51]. Furthermore, numerous genes associated with the SAR process were observed to exhibit increased transcription, including *GRX480*, *TIFY 10-B*, *VDAC1*, *ELI3-2*, *BBE22*, *ALD1*, and the SAR markers *PR1a*, *PR1b*, and *DMR6* [52,53]. In *Arabidopsis*, *GRX480* and *TIFY 10-B* have been demonstrated to positively regulate the SAR response while simultaneously negatively regulating the JA pathway. This indicates that the SAR response was positively enhanced by *MaWRKY45* overexpression. Furthermore, a homolog of the indole-3-acetic acid-amido synthetase gene (*GH3.1*) was observed to be highly up-regulated. The enzyme GH3.1 inactivates auxin, a phytohormone that plays a crucial role in plant growth and development. This process favors the SA or JA defense phytohormone pathways [54], indicating that there is cross-talk between the activation of defense mechanisms and plant growth and development processes. This cross-talk enables the plant to switch between development and defense [55].

Furthermore, we examined the transcriptional profile of TFs to enhance our comprehension of the dynamic transcription process orchestrated by *MaWRKY45* (see Figure 5 and Figure 6). A total of 127 differentially expressed transcription factors were identified. The most representative families were AP2/ERF, WRKY, MYB, bHLH, and HB. It is noteworthy that the genes for MYB TFs exhibited the greatest up-regulation in relation to biotic stress responses. MYB5 (22.5-fold) and MYB14 (22-fold) are involved in the production of secondary metabolites with antimicrobial activity, such as proanthocyanidins (flavonoids). Additionally, MYB14 is associated with the production of stilbenes (phytoalexins), while MYB92 (9-fold) is involved in enhancing suberin deposition in the plant cell wall [56,57,58]. Therefore, these findings indicate that the banana gene *MaWRKY45* is involved in the promotion of antimicrobial compound production in *N. benthamiana* leaves. Conversely, members of the WRKY family are well known for their collaborative regulatory dynamics, with genes belonging to the same family [59]. It was therefore anticipated that a number of WRKY family members would be regulated by the overexpression of *MaWRKY45* in *N. benthamiana*. A phylogenetic tree was thus constructed to observe the clustering pattern of the 10 WRKY DEGs with the 74 *Arabidopsis* WRKY family members and the banana *MaWRKY45* and rice *OsWRKY45* TFs (Figure 7). It is noteworthy that the *N. benthamiana* sequence C90334_g1_i2 was found to be closely related to *Arabidopsis AtWRKY29*, which has been demonstrated to express a phloem mobile cell-to-cell mRNA under nutrient-limited conditions [60] and has been reported as a PTI- [61] and an ISR-inducible gene [62]. Additionally, the WRKY gene c24977_g1_i1 was found to be up-regulated by a factor of five. As illustrated in the phylogenetic tree, this *N. benthamiana* WRKY gene is situated within the same cluster as *AtWRKY70* and *MaWRKY45*. These *N. benthamiana* WRKY genes are part of the same evolutionary clade (Group III) and are associated with biotic stress responses in plants via the regulation of SA and JA crosstalk [14,63]. The observed induction of c24977_g1_i1 transcription may be influenced indirectly by MaWRKY45 TF or directly through its promoter. This pattern of transcriptional regulation, involving either self-autoregulation or cross-regulation among WRKY family members, is commonly observed in this family [64]. It can be inferred that these 127 TFs likely contribute to the significant transcriptional changes mediated by *MaWRKY45*.

It is noteworthy that only one study has been conducted on the downstream genes regulated by *OsWRKY45* [28]. These authors identified 260 DEGs, 220 up- and 40 down-regulated, using microarray analysis in the Nipponbare cultivar and two *WRKY45*-knockdown transgenic lines. The WRKY family of transcription factors was notably represented, including *OsWRKY55*, *OsWRKY62*, *OsWRKY71*, and *OsWRKY72*. Additionally, other *OsWRKY45*-dependent transcription factors, including *OsMYB4*, *OsMYB8*, *OsNAC4*, and *OsHSF1*, were identified. Our own DEG analysis yielded comparable results, with the WRKY and MYB families being among the most represented. However, Nakayama et al. [28] reported only one down-regulated AP2/ERF transcription factor, whereas our analysis identified the AP2/ERF family as the most overrepresented. This discrepancy is probably explained by the fact that their study was limited to OsWRKY45-dependent benzothiadiazole (BTH)-responsive genes. Furthermore, genes such as those encoding cytochrome P450, glutathione S-transferase (GST) (P450 94A1), glutaredoxin (GRX480), chitinases, cellulose synthases, and various membrane and intracellular receptors, which are analogous to those identified in our transcriptomic analysis, were also identified by Nakayama et al. [28] as up-regulated genes. The comprehensive nature of our RNA-seq analysis provided more information than was obtained from the microarray analysis of rice [28]. Nevertheless, notable parallels were identified, indicating that *MaWRKY45* may regulate the response to biotic stress in a manner analogous to that observed in rice. Based on the DEG analysis, we proposed a model that highlights the key players associated with the overexpression of the banana *MaWRKY45* gene in *N. benthamiana* leaves (Figure 8). Considering the common evolutionary origin of the immune system in monocots and dicots [65,66], we expect that the results of the present study using *N. benthamina* as a model system will be similar in banana and other crops. The stable transformation of banana and potentially other crops with *MaWRKY45* under the regulation of an inducible promoter will shed light on this hypothesis.

This study is the first to implement the Infiltration-RNAseq method for the analysis of genes that are directly or indirectly regulated by biotic stress-related TFs in plants. Our findings demonstrate that this is a rapid and convenient method for profiling TF-mediated gene expression changes in *N. benthamiana*, as previously reported by Bond et al. [23]. We were aware that the infiltration of *Agrobacterium* in *N. benthamiana* leaves can lead to both biotic and abiotic stress responses [23]; however, we reasoned that the absence of *WRKY* genes in *Agrobacterium* and the overexpression of a *WRKY* gene such as *MaWRKY45* could cause significant differences in gene expression between *N. benthamiana* plants agroinfiltrated with the construct pCAMBIA2200::*MaWRKY45* or with the empty vector only (mock). Accordingly, the DEGs identified in this study were attributed to the overexpression of *MaWRKY45* in *N. benthamiana* leaves. It was essential to select an appropriate control to observe the effect of overexpressing a biotic stress-related TF in the agroinfiltration assay. The transcriptomic analysis was validated by examining the expression profiles of eight DEGs using RT-qPCR. The expression profiles of all genes demonstrated consistent similarity between the two analyses, thereby reinforcing the reliability of the findings. It is proposed that the Infiltration-RNAseq method offers a robust approach for elucidating the functional roles of biotic stress-related TFs using *N. benthamiana* as a model system.

Our findings suggest that MaWRKY45 TF regulates a diverse set of genes involved in plant immunity, providing a foundation for its application as a biotechnological tool to address key challenges in banana production, including *P*. *fijiensis* and Foc, which threaten banana production worldwide.

## 4. Materials and Methods

### 4.1. Biological Material

The study employed five-week-old *N. benthamiana* plants. The plants were cultivated in individual pots containing sterile peat moss and vermiculite (1:1) under controlled environmental conditions. The plants were grown under a 12 h light/12 h dark cycle, with a light intensity of 100 μmol m^−2^ s^−1^, a temperature of 25 ± 1 °C, and a relative humidity of approximately 70%. The *Escherichia coli* strain DH10B and *Agrobacterium tumefaciens* strain LBA4404 were employed for the purposes of cloning and agroinfiltration, respectively.

### 4.2. Amplification and Cloning of MaWRKY45

The coding sequence (CDS) of the *MaWRKY45* cDNA [18] was amplified using the Expand Long Template PCR System (Roche, Basel, Switzerland) and cloned into the pCAMBIA2200 expression vector, between the CaMV35S promoter and NOS terminator. The cloning was verified by enzymatic digestion and sequencing using the Sanger method (Macrogen Inc., Seoul, Republic of Korea). The *A. tumefaciens* LBA4404 cells were transformed by heat shock treatment with the pCAMBIA2200::MaWRKY45 construct or the empty vector pCAMBIA2200.

### 4.3. Agroinfiltration of N. benthamiana Leaves

The agroinfiltration of *N. benthamiana* leaves was performed in accordance with the methodology outlined by Norkunas et al. [67]. The *A. tumefaciens* cultures with the recombinant DNA vectors were adjusted to an OD600 of 1.0 for leaf infiltration. Two intermediate leaves, situated in the third and fourth positions from the youngest leaf of five-week-old *N. benthamiana* plants, were infiltrated with the pCAMBIA2200::MaWRKY45 construct or the empty vector pCAMBIA2200. A total of 18 *N. benthamiana* plants were utilized for agroinfiltration, with nine plants inoculated with the pCAMBIA2200::MaWRKY45 construct and nine plants inoculated with the pCAMBIA2200 vector. A completely randomized design was employed for the agroinfiltration experiment. After three days, the agroinfiltrated leaves were harvested and stored at −80 °C.

### 4.4. RNA Isolation and cDNA Library Preparation for Sequencing

Total RNA from agroinfiltrated leaves was isolated from 100 mg of tissue using the RNeasy Plant Minikit kit, following the instructions provided by the manufacturer (QIAGEN, Aarhus, Denmark). The concentration of the RNA was determined using a spectrophotometer (NanoDrop™ Lite, Thermo Fisher Scientific, Waltham, MA, USA). Subsequently, we implemented RNA pooling for RNA-seq, in accordance with the recommendations set forth by Assefa et al. [68]. RNA samples from three agroinfiltrated plants, treated with either pCAMBIA2200::*MaWRKY45* or pCAMBIA2200 (mock), were pooled to generate a biological replicate. This process resulted in the generation of a total of six biological replicates for subsequent analysis. The RNA samples were sent to Macrogen Inc. (Seoul, Republic of Korea) for sequencing. The integrity of the RNA was evaluated using the 2100 Bioanalyzer system (Agilent, Santa Clara, CA, USA). Six libraries were generated, three from pCAMBIA2200::MaWRKY45 and three from pCAMBIA2200. The TruSeq Stranded mRNA Library Prep Kit (Illumina, San Diego, CA, USA) was utilized for library construction in accordance with the manufacturer’s instructions. The six libraries were sequenced using the Illumina NovaSeq6000 platform, resulting in 100 bp paired-end reads. The quality of the raw reads was assessed through FASTQC analysis [69].

### 4.5. De Novo Transcriptome Assembly and Functional Annotation

Illumina adapters and reads shorter than 36 bp were removed using Trimmomatic [70]. A de novo transcriptome assembly was conducted using the Trinity v20140717 software [71]. Contigs were filtered and classified as a non-redundant transcript through the use of the CD-HIT-EST program [72]. Open reading frame (ORF) prediction was performed via the TransDecoder v3.0.1 software (https://github.com/TransDecoder/TransDecoder, accessed on 21 January 2022). Curated reads were aligned to the reference assembly using the Bowtie2 software (v2.2.6) [73]. For the functional annotation of unigenes, the sequences were searched against the Kyoto Encyclopedia of Genes and Genomes (KEGG) database v20220103 [74] (http://www.genome.jp/kegg/ko.html, accessed on 21 January 2022), NCBI Nucleotide (NT) v20180116 [75] (https://www.ncbi.nlm.nih.gov/nucleotide/, accessed on 21 January 2022), Pfam v20160316 [76] (http://pfam.xfam.org/, accessed on 21 January 2022), Gene Ontology (GO) v20180319 [77] (http://www.geneontology.org/, accessed on 21 January 2022), NCBI non-redundant Protein (NR) v20180503 [78] (https://www.ncbi.nlm.nih.gov/protein/, accessed on 21 January 2022), UniProt v20180116 [79] (http://www.uniprot.org/, accessed on 21 January 2022), and EggNOG veggnog4 [80] (http://eggnogdb.embl.de/, accessed on 21 January 2022) using BLASTN from the National Center for Biotechnology Information (NCBI) and BLASTX [81] from the DIAMOND software v0.9.21 [82] with an E-value cutoff of 1 × 10^−5^.

### 4.6. Analysis of Differentially Expressed Genes (DEGs)

The abundance of unigenes in each sample was estimated as read counts, a measure of expression, using the RSEM algorithm [83]. The differential expression analysis of the genes was conducted by comparing the data from samples transiently overexpressing *MaWRKY45* in *N. benthamiana* leaves (Nb-W45-OE1, Nb-W45-OE2, and Nb-W45-OE3) against three mock samples transiently transformed with the empty vector (Nb-EV1, Nb-EV2, and Nb-EV3). The identification of DEGs was conducted using the DESeq2 method, as described by Love et al. [84]. Transcript abundance was expressed in fragments per kilobase of exon model per million mapped reads (FPKM). The identification of DEGs was based on two criteria: a false discovery rate (FDR) of less than 0.05 and a fold change (FC) of at least 2. The DEGs were subjected to GO term pathway enrichment analyses using Fisher’s exact test with a cutoff of false discovery rate (FDR) < 0.05. The GO enrichment data and bar graphs were plotted using the ggplot2 v.3.5.1 package [85] in RStudio v.4.4.2, which is designed for data analysis and visualization. A search for differentially expressed TFs responsive to various forms of stress (bacteria, fungi, viruses, and phytohormones) was conducted through data mining using keywords in the GO database, which had previously been generated for gene functional annotation and gene expression profiling. The resulting DEGs were subsequently visualized through heat map graphs generated by the ComplexHeatmap v.2.20.0 package in RStudio v.4.4.2 [86]. A phylogenetic tree was constructed using 10 WRKY DEGs from *N. benthamiana* leaves overexpressing the banana gene *MaWRKY45* and the complete set of WRKY family members from *Arabidopsis thaliana*, plus *MaWRKY45* and *OsWRKY45*. The protein sequences were aligned using the MUSCLE algorithm, and a phylogenetic tree was constructed using the neighbor-joining (NJ) method with 1000 bootstrap replications. This was performed employing the Molecular Evolutionary Genetics Analysis (MEGA) v11.0.10 package [87] (http://www.megasoftware.net/, accessed on 13 April 2024). The phylogenetic tree was edited using the FigTree v1.4.4 program (http://tree.bio.ed.ac.uk/software/figtree, accessed on 8 June 2024).

### 4.7. RT-qPCR

Total RNA was extracted from the same *N. benthamiana* leaf material used for the Infiltration-RNAseq assay. cDNA synthesis was carried out from 5 µg of total RNA using the SuperScript III Reverse Transcriptase (Thermo Fisher Scientific). Oligonucleotides were designed for eight DEGs (Table S5) and the elongation factor 1α (EF1α) from *N. benthamiana* [88] was used as a reference gene. A real-time quantitative polymerase chain reaction (RT-qPCR) was conducted using the StepOnePlus™ Real-Time PCR System (Thermo Fisher Scientific). The program utilized a starting temperature of 95 °C for 10 min, followed by 40 cycles of 95 °C for 10 s, 57 °C for 30 s, and 72 °C for 30 s. The relative expression was calculated using the 2^−ΔΔCt^ method [25]. A statistical analysis was conducted using three biological replicates, as previously described. Each biological replicate comprises the total RNA pooling of three independent *N. benthamiana* plants under the same treatment (*MaWRKY45* overexpression or mock). The Anderson–Darling statistic was utilized to assess the normality assumption for a *t*-test. Subsequently, a Student’s *t*-test was employed via Minitab software v.17 (Minitab Inc., State College, PA, USA).

## 5. Conclusions

Overexpression of *MaWRKY45* in *N. benthamiana* leaves significantly altered the expression of genes involved in plant immune responses. In particular, numerous genes that were up-regulated encoded proteins that were homologs to disease resistance receptors, proteins involved in cell wall reinforcement, proteins with antimicrobial activity, and transcription factors that are related to plant immunity. The Infiltration-RNAseq method thus demonstrated that the banana *MaWRKY45* encodes a functional TF capable of regulating the expression of multiple genes related to plant immunity. These insights reinforce the notion that *MaWRKY45* is a promising candidate for bioengineering disease resistance in banana using, for instance, a pathogen-inducible promoter. Moreover, the Infiltration-RNAseq method, which employs *N. benthamiana* as a model plant, could facilitate and expedite the functional analysis of defense-related TFs from tropical crops, where the agroinfiltration technique is inapplicable or the generation of stable transgenic plants is a lengthy process.

## Figures and Tables

**Table 1:** Statistics of de novo transcriptome assembly of *N*. *benthamiana*.

Metric	Transcript Contig	Only Longest Isoform per Gene
N50 (bp)	1289	898
Average contig length (bp)	782	620
Maximum contig length (bp)	14,833	14,833
Minimum contig length (bp)	201	201
GC content (%)	38	38
Total transcripts	273,793	189,020

## Data Availability

Original RNA-seq data are openly available in Gene Expression Omnibus under accession GSE282538.

## Supplementary Materials

The following supporting information can be downloaded at https://www.mdpi.com/article/10.3390/plants14030483/s1: Figure S1: RNA integrity and MaWRKY45 RT-PCR detection. (a) Total RNA from three biological control replicates and three biological replicates with the MaWRKY45 expression construct were fractionated on 1.5% agarose gel and stained with ethidium bromide. (b) PCR amplification of the MaWRKY45 CDS. Total RNA was isolated from all biological replicates of N. benthamiana leaves, cDNA was synthesized, and PCR reactions were performed using specific primers to detect the CDS of the MaWRKY45 banana gene (888 bp in length). Lanes 1–3 represent the three mock replicates (Nb_EV_1-3 samples), while lanes 4–6 represent the three biological replicates overexpressing the MaWRKY45 gene (Nb_W45-OE_1-3 samples); Figure S2: Expression analysis after 3 days of agroinfiltrating samples with the empty vector and overexpressing the MaWRKY45 CDS. (a) Correlation analysis between samples, where the X and Y axes represent each sample. The color represents the correlation coefficient (a darker color indicates higher correlation). (b) Hierarchical clustering among samples. The closer samples indicate similar expression levels. (c) Gene expression boxplot. The X-axis represents the sample name. The Y-axis represents the log10FPKM value. (d) Gene expression density map. The X-axis represents the log10FPKM value. The Y-axis represents the gene density; Table S1: Quality control measures of total RNA. Table S2: Summary of quality control metrics for sequencing libraries. Table S3: Statistics of filtered sequence data. Table S4: Differentially expressed genes with the highest fold change values; 60 genes with the highest fold change values (30 positively regulated and 30 negatively regulated) are represented. DEGs are defined as those with a fold change (FC) ≥ 2 and a Benjamini–Hochberg FDR-corrected *p*-value < 0.05. Table S5: Primer sequences for RNAseq validation of 10 differentially expressed genes.
