# Supplementary material for: Infiltration-RNAseq Reveals Enhanced Defense Responses in Nicothiana benthamiana Leaves Overexpressing the Banana Gene MaWRKY45
Source: Plants (Basel). 2025 Feb 6;14(3):483. doi: 10.3390/plants14030483 (PMC11820619; doi:10.3390/plants14030483)
Supplement: Supplementary file 1 [file plants-14-00483-s001.zip › plants-3357943-supplementary.pdf]

Supplementary Materials

Infiltration-RNAseq reveals enhanced defense responses in *Nicothiana benthamiana* leaves overexpressing the banana *MaWRKY45* gene

Sergio Garcia-Laynes[1], Carlos Ligne Calderón-Vázquez[2], Carlos Puch-Hau[3], Virginia Aurora Herrera-Valencia[1]*, and Santy Peraza-Echeverria[1]*

1 Unidad de Biotecnología, Centro de Investigación Científica de Yucatán, Calle 43 No. 130 × 32 y 34, Colonia Chuburná de Hidalgo, Mérida 97205, Yucatán, Mexico

2 Instituto Politécnico Nacional, CIIDIR Unidad Sinaloa, 81100 Guasave, Sinaloa, Mexico

3 Tecnológico Nacional de México Campus Instituto tecnológico Superior de Valladolid, Carretera Valladolid-Tizimín, km 3.5, C.P. 97780, Valladolid, Yucatán, México

* Correspondence: SPE: santype@cicy.mx ; VAHV vicky@cicy.mx; Tel.: +52-9999-428-330 ext. 202

Supplementary material

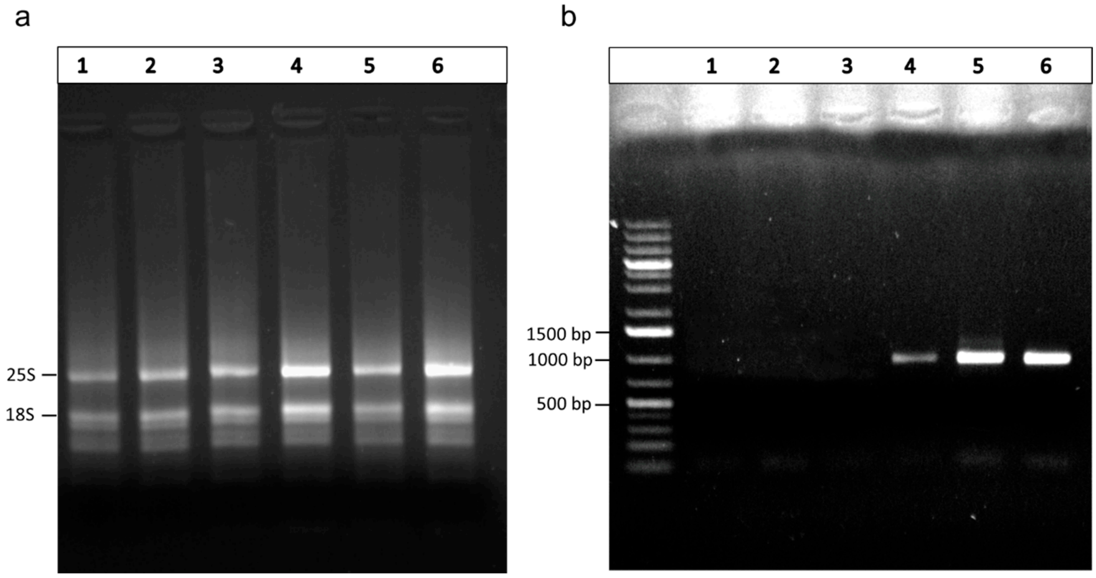

Figure S1. RNA integrity and *MaWRKY45* RT-PCR detection. a) Total RNA from three biological control replicates and three biological replicates with the *MaWRKY45* expression construct were fractionated on a 1.5% agarose gel and stained with ethidium bromide. b) PCR amplification of the *MaWRKY45* CDS. Total RNA was isolated from all biological replicates of *N. benthamiana* leaves, cDNA was synthesized, and PCR reactions were performed using specific primers to detect the CDS of the *MaWRKY45* banana gene (888 bp in length). Lanes 1-3 represent the three mock replicates (Nb_EV_1-3 samples), while lanes 4-6 represent the three biological replicates overexpressing the *MaWRKY45* gene (Nb_W45-OE_1-3 samples).

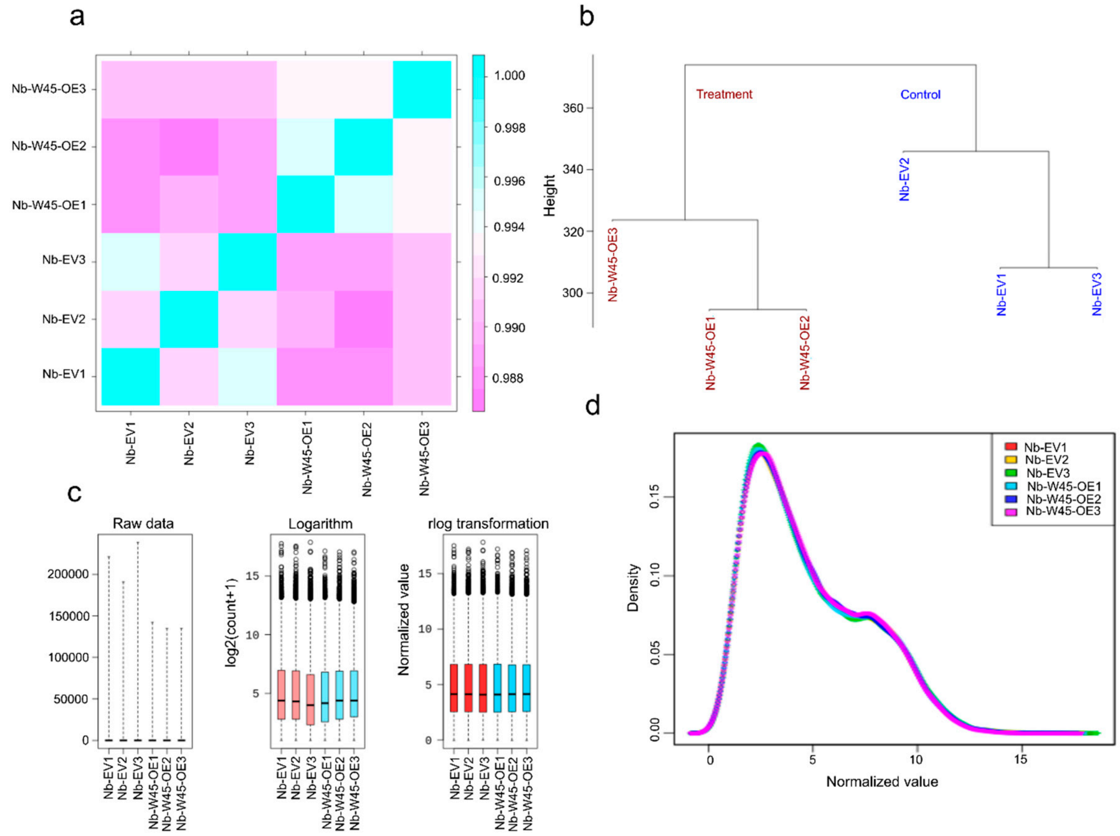

Figure S2. Expression analysis after 3 days of agroinfiltrating samples with the empty vector and overexpressing the *MaWRKY45* CDS. a) Correlation analysis between samples. Where, the X and Y axes represent each sample. The color represents the correlation coefficient (darker color indicates higher correlation). b) Hierarchical clustering among samples. The closer samples indicate similar expression levels. c) Gene expression Boxplot. The X-axis represents the sample name. The Y-axis represents the log10FPKM value. d) Gene expression density map. The X-axis represents the log10FPKM value. The Y-axis represents the gene density."

Table S1. Quality control measures of total RNA.

Sample	260/280 ratio	RIN
Nb-W45-OE1	2.06	8
Nb-W45-OE2	2.07	7.8
Nb-W45-OE3	2.06	7.7
Nb-EV1	2.06	7.1
Nb-EV2	2.06	7.5
Nb-EV3	2.07	8.8

* Nb-WRKY45-OE and Nb-EV stand for *N. benthamiana WRKY45* Overexpression and *N. benthamiana* Empty Vector (mock), respectively. The numbers 1, 2 and 3 indicate the biological replicate number.

*RIN, stands for RNA Integrity Number.

Table S2. Summary of quality control metrics for sequencing libraries.

Library name	Library type	Concentration (ug/ul)	Concentration (nM)	Size (bp)
Nb-W45-OE1	TruSeq stranded mRNA	16.8	75.3	320
Nb-W45-OE2	TruSeq stranded mRNA	21.8	94.1	327
Nb-W45-OE3	TruSeq stranded mRNA	12.6	52.7	342
Nb-EV1	TruSeq stranded mRNA	6.82	29.8	324
Nb-EV2	TruSeq stranded mRNA	15.8	68	325
Nb-EV3	TruSeq stranded mRNA	9	39.6	320

* Nb-WRKY45-OE and Nb-EV stand for *N. benthamiana WRKY45* Overexpression and *N. benthamiana* Empty Vector (mock), respectively. The numbers 1, 2 and 3 indicate the biological replicate number.

Table S3. Statistics of filtered sequence data.

Sample	Clean reads	GC %	Q20(%)	Q30(%)	# of mapped reads
Nb-WRKY45-OE1	48,964,020	42.63	98.81	95.76	29,466,854 (60.18%)
Nb-WRKY45-OE2	52,367,310	42.48	98.74	95.57	31,507,486 (60.17%)
Nb-WRKY45-OE3	49,668,180	42.23	98.76	95.59	29,540,364 (59.48%)
Nb-EV1	55,172,720	42.42	98.86	95.92	33,096,048 (59.99%)
Nb-EV2	55,770,394	43.13	98.84	95.79	32,833,156 (58.87%)
Nb-EV3	45,366,776	42.50	98.74	95.52	27,811,088 (61.3%)
Total	307,309,400				184,254,996

* Nb-WRKY45-OE and Nb-EV stand for *N. benthamiana WRKY45* Overexpression and *N. benthamiana* Empty Vector (mock), respectively. The numbers 1, 2 and 3 indicate the biological replicate number.

Table S4. DEGs with the highest fold change values are shown. 60 genes with the highest fold change values (30 positively regulated and 30 negatively regulated) were represented. DEGs were defined as those with a fold change (FC) ≥ 2 and a Benjamini-Hochberg FDR-corrected p-value < 0.05.

#	Gene ID	Gene name	Protein activity	Subcellular Localization	Biological process	Fold_change
			**UP REGULATED GENES**			
1	c86039_g1_i1	Nudix hydrolase 2-like	hydrolase activity	Cytoplasm	Response to oxidative stress	**33.004058**
2	c54821_g1_i1	Trypsin inhibitor 1-like (potato_inhibit)	Peptidase activity	Extracellular	Defense response to fungus/response to wounding	**31.889631**
3	c98684_g2_i1	NRT1/ PTR FAMILY 7.3-like	transporter activity	Membrane	Response to nitrate	**29.415224**
4	c74390_g1_i1	Keratin, type I cytoskeletal 9-like	-	-	-	**22.870795**
5	c95394_g4_i4	Transcription repressor MYB5-like	Transcription factor activity	Nucleus	Seed germination/trichrome differentiation	**22.573151**
6	c6266_g1_i1	MYB14	Transcription factor activity	Nucleus	cell differentiation/response to salt/response to freezing	**22.053513**
7	c82241_g2_i1	subtilisin-like protease SBT1.7	serine-type endopeptidase activity	Cell wall	-	**21.327421**

8	c121351_g1_i1	indole-3-acetic acid-amido synthetase (GH3.1)	Ligase activity	Cytoplasm	Response to auxin	**20.119373**
9	c67623_g1_i2	Uncharacterized	-	-	-	**18.476865**
10	c88334_g2_i1	Cationic peroxidase 1-like	Peroxidase activity	Extracellular region	Response to oxidative stress	**16.826092**
11	c50214_g1_i1	Uncharacterized	-	-	-	**16.374693**
12	c98553_g2_i4	Microtubule-associated protein 70-5-like	Microtubule binding	Cytoplasm	Cell wall organization	**15.663553**
13	c76634_g2_i3	bHLH162-like	Transcription factor activity	Nucleus	-	**15.615904**
14	c99197_g1_i1	Beta-D-xylosidase 1-like	Xylan 1,4-beta-xylosidase activity	Cell wall	Xylan catabolic process	**15.464113**
15	c75853_g2_i1	Branched-chain-amino-acid aminotransferase 2	Catalytic activity	Cytoplasm	Amino acid biogenesis	**15.382905**
16	c95261_g7_i4	Uncharacterized peptidase	Peptidase activity	-	-	**15.288214**
17	c84310_g1_i1	CONSTANS-LIKE 2-like	Transcription factor activity	Nucleus	Regulation of flower development	**15.148669**
18	c70942_g3_i1	Chaperone protein dnaJ 20	Folding	Chloroplast	-	**15.036430**
19	c93307_g26_i1	Probable disease resistance protein (HR2)	-	-	-	**14.722448**
20	c89138_g2_i4	Probable serine/threonine-protein kinase WNK4	Protein serine/threonine kinase activity	Cytoplasm	Intracellular signal transduction	**14.555279**
21	c95764_g2_i1	Protein IQM3	Calmodulin binding	Cytoplasm/ Nucleus/Chloroplast	-	**14.291853**
22	c93342_g8_i1	Inositol 2-dehydrogenase-like	Oxidoreductase activity	-	Oxidation-reduction process	**14.083394**
23	c54440_g1_i1	Cation/H(+) antiporter 18-like	Proton antiporter activity	-	Regulation of pH	**14.070267**
24	c66466_g1_i1	histone H2B-like	-	-	-	**12.445786**
25	c89071_g1_i2	Heavy metal-associated isoprenylated plant protein 20-like	Metal ion transport	Cytoplasm	Cellular transition metal ion homeostasis	**12.078625**
26	c2624_g2_i1	xyloglucan endotransglucosylase/ hydrolase protein 16	Hydrolase activity	Cell wall	Cell wall biogenesis	**11.791959**
27	c63510_g1_i1	Aluminum-activated malate transporter 2	Malate transmembrane transporter activity	Vacuole membrane		**11.324505**
28	c44491_g1_i1	F-box/kelch-repeat protein	Ubiquitin-protein transferase activity	-	Proteasomal catabolic process	**11.232088**
29	c50869_g1_i1	Uncharacterized	Integral component of mitochondria membrane	Mitochondria	Defense response to fungus	**11.060097**
30	c91935_g1_i1	Flavin-containing monooxygenase	monooxygenase activity	-	Oxidation-reduction process	**10.846860**

DOWN REGULATED GENES

31	c22778_g2_i1	peroxidase 70-like	Peroxidase activity	Extracellular region	Response to oxidative stress	**-146.692552**
32	c79432_g1_i2	Calvin cycle protein CP12-2	Protein binding	Chloroplast	Reductive pentose-phosphate cycle	**-121.782986**
33	c22778_g1_i1	Cationic peroxidase 1-like	Peroxidase activity	Extracellular region	Pesponse to oxidative stress	**-83.490394**
34	c91200_g1_i1	peroxidase 5-like	Peroxidase activity	Cell wall	Plant-type cell wall organization	**-69.003335**
35	c84135_g2_i1	MYB36	Transcription factor activity	nucleus	cell differentiation	**-57.558788**
36	c60482_g1_i1	Proline-rich extensin-like protein EPR1	-	-	-	**-41.462491**
37	c73290_g2_i1	Serine/threonine-protein kinase HT1-like	-	-	-	**-39.447026**
38	c151802_g1_i1	LTP_2	Lipid binding	Extracellular region	Lipid transport	**-39.286353**
39	c80309_g1_i4	Glutamate decarboxylase-like	Pyridoxal phosphate binding	Cytoplasm	Glutamate metabolic process	**-36.037157**
40	c94450_g1_i2	Patellin-4-like	Transporter activity	Nucleus/plasma membrane/cytoplasm	cell cycle	**-27.429015**
41	c75199_g1_i4	Vesicle transport protein GOT1B-like	Vesicle-mediated transport	Plasma membrane	Vesicle-mediated transport	**-25.375719**
42	c49131_g1_i2	Lipid-transfer protein-like protein	Lipid binding	Plasma membrane	Lipid transport	**-24.182679**
43	c91627_g2_i1	Putative germin-like protein 2-1	Oxalate decarboxylase activity	Cell wall	Oxalate metabolic process	**-22.590016**
44	c74581_g1_i1	Cysteine protease inhibitor 8-like	-	Vacuole	-	**-21.546766**
45	c92242_g1_i1	Putative two-component response regulator-like APRR6	-	-	-	**-18.374051**
46	c96280_g6_i1	Histone deacetylase 2-like	Histone deacetylase activity	Nucleus	Regulation of transcription	**-18.083386**
47	c29479_g2_i1	3-ketoacyl-CoA synthase 6-like	Integral component of membrane	Endoplasmic reticulum membrane	Fatty acid biosynthetic process	**-17.883296**
48	c94012_g4_i8	Uncharacterized	Integral component of membrane	Plasma membrane	Integral component of membrane	**-17.003308**
49	c137410_g1_i1	Stress-inducible protein TAS14-like	-	-	-	**-16.975060**
50	c100522_g5_i1	Amino acid permease 6-like	amino acid transmembrane transporter activity	Plasma membrane	Amino acid transmembrane transport	**-16.533300**
51	c97639_g2_i2	ABI five binding protein 2	Protein binding	Nucleus	response to water deprivation/ Signal transduction	**-16.055005**
52	c96544_g2_i3	Transmembrane 9 superfamily member 11-like	integral component of membrane	Plasma membrane	integral component of membrane	**-16.048396**

53	c94029_g1_i1	Vacuolar cation/proton exchanger 3-like	Calcium:cation antiporter activity	Vacuole	Cellular calcium ion homeostasis	**-16.030373**
54	c29831_g1_i1	Defensin-like protein 19	-	-	-	**-15.675318**
55	c97101_g1_i5	ABSCISIC ACID-INSENSITIVE 5-like	Transcription factor activity	Nucleus	water deprivation/salt	**-15.653345**
56	c136888_g1_i1	Protein LE25-like	-	-	Embryo development ending in seed dormancy/response to cold and water deprivation	**-15.607630**
57	c78801_g4_i1	Defensin-like protein 1	-	-	-	**-14.977649**
58	c96101_g1_i5	Zinc finger Ran-binding domain-containing protein 2-like	-	Nucleus	-	**-13.794202**
59	c87626_g1_i3	Uncharacterized	Beta-glucosidase activity	-	Response to salt stress	**-13.195476**
60	c91874_g1_i1	HB-12	Transcription factor activity	Nucleus	Response to virus, salt and water deprivation	**-12.562572**

Table S5. Primer sequences for RNAseq validation of 10 genes differentially expressed.

ID	Gen name	RNASeq Fold Change	Primer sequence (5'->3')
c84135_g2_i1	MYB36	-57	Forward: gctctccctcaaaaagcagg<br>Reverse: ttgcagctatcacagaccacc
c75199_g1_i4	GOT1B-like	-25	Forward: ccattggagtgatgcctacc<br>Reverse: gccaaaagccactaaacagc
c97639_g2_i2	ABI	-16	Forward: gagttgtcggaagcagaaca<br>Reverse: tgtcttccccccttaccatact
c91874_g1_i1	HB-12	-12	Forward: cgtcaggttgcaatttggtt<br>Reverse: tgtccaccttctttctcgga
c86039_g1_i1	Nud2	33	Forward: gttggtattggtgctctcgt<br>Reverse: tgtctgaaggcgaggatttg
c6266_g1_i1	MYB14	22	Forward: acatgtgaagtgatggatgttgtg<br>Reverse: gtgatcaaactgatgacaaagttga
c76634_g2_i3	bHLH162-like	15	Forward: agaaatccacactatggattcagc<br>Reverse: ggaaagaaacaaatggaaaaccttag
c92405_g2_i1	BBE22	8	Forward: agtacttggtcaactggggt<br>Reverse: ccccagattttggcctttga
NbEF1α Reference gene (Zhang et al., 2023)	NbEF1α	-	Forward: agctttacctcccaagtcatc<br>Reverse: agaacgcctgtcaatcttgg
